# High-flow nasal oxygen in perioperative and critical care: a bibliometric analysis

**DOI:** 10.3389/fmed.2026.1856380

**Published:** 2026-07-15

**Authors:** Biao Xi, Yuting Zhao, Wei Fu, Yunzhi Ling, Qin Zhuang, Di Zhang

**Affiliations:** 1 Department of Anesthesiology, The First Affiliated Hospital of Bengbu Medical University, Bengbu, China; 2 Department of Burns, The First Affiliated Hospital of Anhui Medical University, Hefei, China

**Keywords:** bibliometric analysis, CiteSpace, critical care, high-flow nasal cannula, high-flow nasal oxygen, perioperative care, science mapping, VOSviewer

## Abstract

**Background:**

High-flow nasal oxygen (HFNO), also known as high-flow nasal cannula, is an important noninvasive respiratory support strategy in perioperative and critical care practice. As the literature has expanded across multiple clinical contexts, a structured overview is needed to clarify the development, knowledge base, and emerging priorities of the field.

**Methods:**

We conducted a bibliometric and visual analysis of HFNO research using English-language articles and reviews retrieved from the Web of Science Core Collection, Scopus, and PubMed for the period 2000–2025. After screening, merging, and deduplication, 2,314 unique publications were included. Bibliometrix in R was used for performance analysis, thematic mapping, and thematic evolution; VOSviewer for collaboration analysis; and CiteSpace for co-citation analysis, keyword clustering, timeline visualization, burst detection, and dual-map overlay.

**Results:**

HFNO research showed sustained exponential growth, with marked acceleration after 2018 and especially after 2020. The literature was concentrated in respiratory medicine, critical care, and anesthesiology journals, with Respiratory Care ranking first in publication output. The United States and China were the leading contributors, while several French institutions, particularly Assistance Publique-Hôpitaux de Paris, were prominent in the institutional network. Co-citation analysis identified major clusters related to acute respiratory failure, perioperative oxygen therapy, preoxygenation strategies, and coronavirus disease. Keyword and thematic analyses indicated a shift from early emphasis on perioperative oxygenation and postoperative respiratory support toward broader critical care application and, more recently, toward context-specific deployment, acute hypoxemic respiratory failure, awake prone positioning, treatment monitoring, and the ROX index.

**Conclusion:**

HFNO research has evolved from a focused literature on oxygenation support into a broader and more clinically differentiated field spanning perioperative and critical care practice. Current hotspots are increasingly centered on context-specific use, monitoring, failure prediction, and escalation decisions. Future research should prioritize clinically actionable patient stratification, standardized outcome definitions, and protocol-based integration of HFNO across different care pathways.

## 1 Introduction

High-flow nasal oxygen (HFNO), also known as high-flow nasal cannula (HFNC), has developed from an adjunctive oxygen delivery technique into an established noninvasive respiratory support modality in both perioperative and critical care practice. By delivering heated and humidified gas at high flow rates with precise control of the inspired oxygen fraction, HFNO can improve oxygenation while maintaining patient comfort and preserving a noninvasive interface (1). Current clinical guidance recognizes HFNO as an important option for selected patients with acute respiratory failure (2).

In critical care, HFNO is widely applied in acute hypoxemic respiratory failure and in clinical situations where oxygenation must be stabilized while treatment response and the need for escalation are closely monitored. Randomized trials and comparative studies have supported its use in these settings (3, 4). Subsequent research has increasingly focused on identifying patients most likely to benefit, recognizing early treatment failure, and refining bedside decision-making. The ROX index reflects this shift. It was initially proposed as a practical predictor of HFNO success in pneumonia-related hypoxemic respiratory failure and was later evaluated in a multicenter validation study (5, 6). Comparative evidence syntheses have also examined HFNO in relation to standard oxygen therapy and other noninvasive oxygenation strategies in adults with acute hypoxemic respiratory failure (4, 7).

In perioperative care, HFNO has received substantial attention for preoxygenation, apneic oxygenation, and oxygenation support during sedation and airway management. Although perioperative and critical care studies partly overlap, these research streams differ in patient populations, procedural contexts, and clinical endpoints. Early work, including the THRIVE approach (transnasal humidified rapid-insufflation ventilatory exchange), showed that nasal high-flow oxygenation could prolong apnea time and improve oxygenation during airway interventions (8). Later studies and reviews further expanded its applications in anesthesia, procedural sedation, and selected high-risk perioperative populations (9, 10). HFNO research has therefore progressed across multiple clinical scenarios rather than along a single unified pathway.

As the literature has grown, the field has become increasingly diverse. HFNO research now encompasses respiratory physiology, noninvasive respiratory support, peri-intubation management, COVID-19-related care, and more specific applications in selected patient groups. Traditional narrative reviews and meta-analyses remain essential for addressing focused clinical questions, but they are less suited to delineating the broader knowledge structure of a rapidly expanding field, including publication trends, influential journals, leading contributors, collaboration patterns, and the evolution of research themes.

Bibliometric analysis offers a complementary approach by quantifying scientific productivity, mapping collaboration and co-citation networks, and identifying emerging topics over time (11–15). In the present study, we conducted a bibliometric and visual analysis of HFNO research in perioperative and critical care settings based on publications retrieved from the Web of Science Core Collection, Scopus, and PubMed. The study aimed to characterize publication growth, identify leading journals and contributors, map collaboration patterns, and reveal the knowledge base, thematic evolution, and emerging hotspots in HFNO research. By doing so, we sought to provide a structured overview of how the field has developed and to clarify its likely future directions.

## 2 Materials and methods

### 2.1 Data source and search strategy

A comprehensive literature search was conducted in the Web of Science Core Collection (WoSCC), Scopus, and PubMed to identify publications on high-flow nasal oxygen (HFNO) in perioperative and critical care settings. The search strategy combined terms related to HFNO and its commonly used alternative terminology, including “high-flow nasal oxygen,” “high-flow nasal cannula,” “HFNO,” “HFNC,” “nasal high-flow,” “high-flow nasal therapy,” “THRIVE,” and “transnasal humidified rapid-insufflation ventilatory exchange,” with terms related to perioperative care, anesthesia, surgery, airway management, intensive care, and critical care. Neonatal and pediatric terms were excluded because these populations differ substantially from adult perioperative and critical care practice in indications, device settings, and care pathways. The search period covered 1 January 2000 to 31 December 2025. Only articles and reviews published in English were included. The search retrieved 1,532 records from WoSCC, of which 1,358 met the predefined eligibility criteria. In Scopus, 1,210 records were initially identified, and 1,126 were retained after application of the same restrictions. In PubMed, 432 records met the eligibility criteria. The complete search strategies for all three databases are provided in the Supplementary Material.

### 2.2 Data screening, merging, and preprocessing

**Figure 1:**
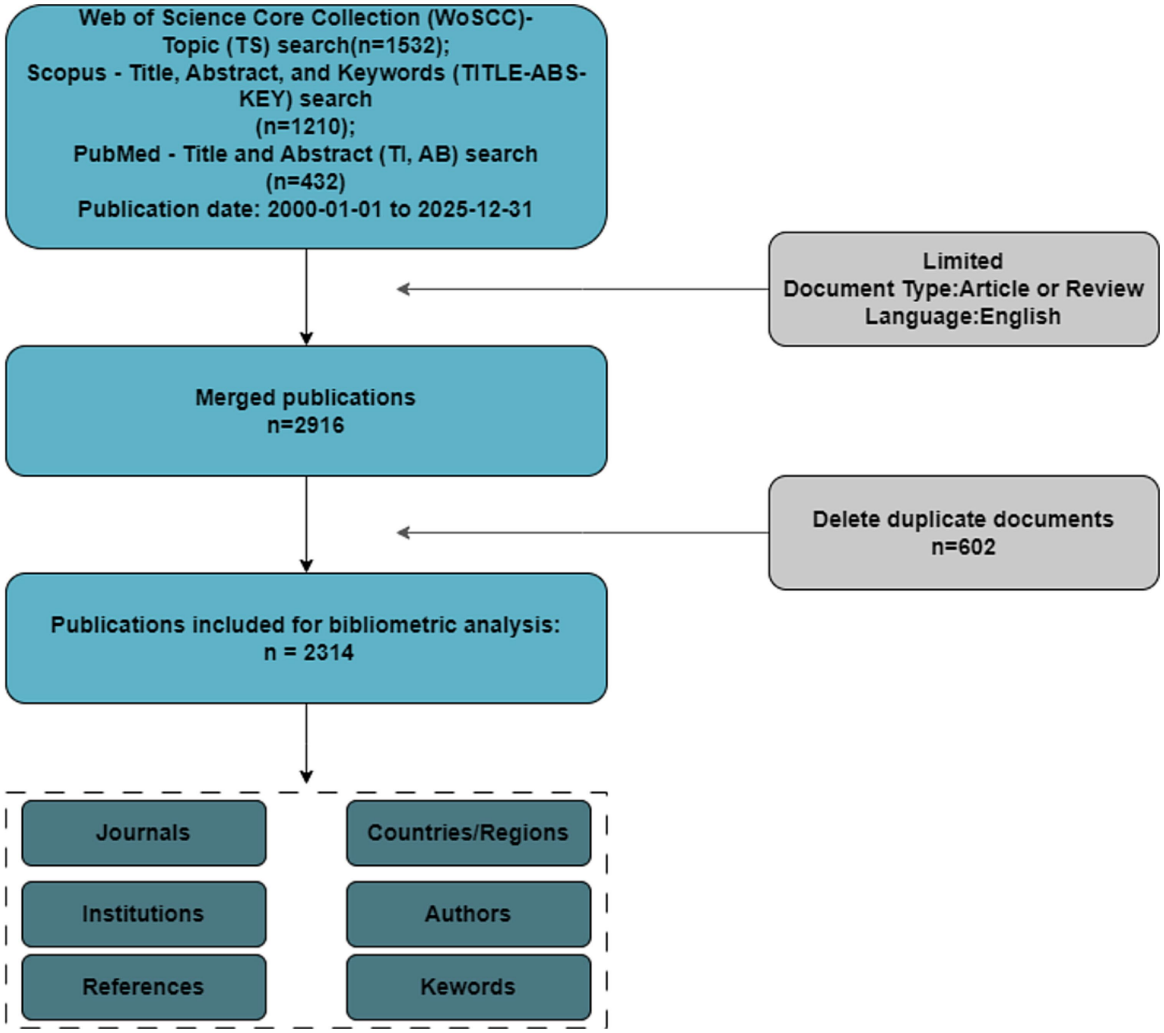
Study workflow for literature retrieval, screening, and preprocessing. Records were retrieved from the Web of Science Core Collection, Scopus, and PubMed. After database-specific screening, 1,358 records from WoSCC, 1,126 from Scopus, and 432 from PubMed were retained. These 2,916 records were merged and deduplicated, leaving 2,314 unique publications for the final bibliometric analysis.

Records retained after database-specific screening were imported into the bibliometrix workflow in R for format conversion, merging, and deduplication (11, 16). Deduplication was performed in a stepwise manner. Records were first matched using unique identifiers, including DOI and PMID when available. For records without complete identifiers, duplicate detection was further based on normalized titles, author names, publication year, journal source, and other bibliographic fields. Potential duplicate or conflicting records were manually checked to reduce erroneous exclusion and to preserve the most complete metadata record. A total of 2,916 records were collected from the three databases, and 2,314 unique publications remained after duplicate removal. The cleaned dataset was used for all subsequent bibliometric analyses. For analyses performed in CiteSpace, records from the three databases were further harmonized into a compatible format before import, allowing co-citation, keyword, and temporal analyses to be conducted within a unified dataset. The screening, merging, and preprocessing procedures are summarized in Figure 1.

### 2.3 Bibliometric indicators and performance analysis

Bibliometric performance was evaluated at the levels of journals, countries, institutions, and authors. The main indicators included number of publications, total citations, average citations per publication, and H-index (17). Core journals were identified according to Bradford’s law (18), and author productivity patterns were interpreted with reference to Lotka’s law (19). Descriptive analyses were mainly performed in R (version 4.5.2) using the bibliometrix package (11, 16). Annual publication output and cumulative growth trends were further visualized in Microsoft Excel, and an exponential fitting curve was applied to characterize long-term publication growth.

### 2.4 Network visualization and science mapping

Collaboration patterns at the country, institution, and author levels were analyzed using VOSviewer (version 1.6.20) (12). In these maps, node size represents publication output or occurrence frequency, link thickness reflects collaboration strength or co-occurrence intensity, and colors indicate clusters identified by the built-in clustering algorithm. Country-level geographic distribution was visualized in R, and selected collaboration maps were further refined in SCImago Graphica to improve layout and visual presentation.

CiteSpace (version 6.4.R2) was used for co-citation analysis, timeline visualization, burst detection, dual-map overlay, and keyword-based analyses to examine the knowledge structure and evolving research trends of HFNO (13, 15). CiteSpace (version 6.4.R2) was used for co-citation analysis, timeline visualization, burst detection, dual-map overlay, and keyword-based analyses to examine the knowledge structure and evolving research trends of HFNO (13, 15). CiteSpace-based analyses were restricted to the period 2015–2025, with 1 year per slice and Top 30 items per slice used as the selection criterion. This time window was selected for both methodological and clinical reasons. Methodologically, the number of publications before 2015 was small, which made time-sliced co-citation and keyword networks less stable and less interpretable. Clinically, 2015 marked an important turning point after the publication of the FLORALI trial, which substantially increased attention to HFNO in acute hypoxemic respiratory failure and helped move the field from physiological interest toward comparative clinical evaluation (3). This setting therefore allowed clearer identification of network structure, cluster patterns, and temporal evolution during the most active phase of HFNO research. Cluster quality was assessed using modularity Q and mean silhouette S values (13, 20). Burst detection was interpreted according to Kleinberg’s burst-detection approach (21), and recent bibliometric studies have likewise used burst analysis to identify research hotspots (22).

Keyword analysis in CiteSpace included keyword clustering timeline mapping and strongest citation burst detection to trace shifts in research focus and identify recent hotspots. In addition thematic mapping and thematic evolution were performed in bibliometrix based on co-word analysis of authors’ keywords to evaluate the centrality development and temporal transition of major themes (11, 14, 16).

### 2.5 Software environment and reproducibility

All analyses were based on a fixed dataset compiled from the three databases within the predefined search period. Data merging, deduplication, descriptive bibliometric analysis, and thematic mapping were conducted in R using bibliometrix within RStudio. Collaboration networks were generated in VOSviewer, with selected layouts refined in SCImago Graphica. Co-citation analysis, keyword clustering, timeline mapping, burst detection, and dual-map overlay were performed in CiteSpace, while Microsoft Excel was used for selected trend visualizations. The use of a single cleaned dataset, together with fixed analytical settings across software platforms, was intended to enhance the transparency and reproducibility of the study.

## 3 Results

### 3.1 Study selection and dataset profile

The literature retrieval and screening process is summarized in Figure 1. After database-specific screening, records from the Web of Science Core Collection, Scopus, and PubMed were merged for further processing. A total of 2,916 records were identified across the three databases. After deduplication, 602 duplicate records were removed, leaving 2,314 unique publications for the final bibliometric analysis. This dataset was used for all subsequent analyses of journals, countries, institutions, authors, references, and keywords.

### 3.2 Publication growth and developmental trend of the field

**Figure 2:**
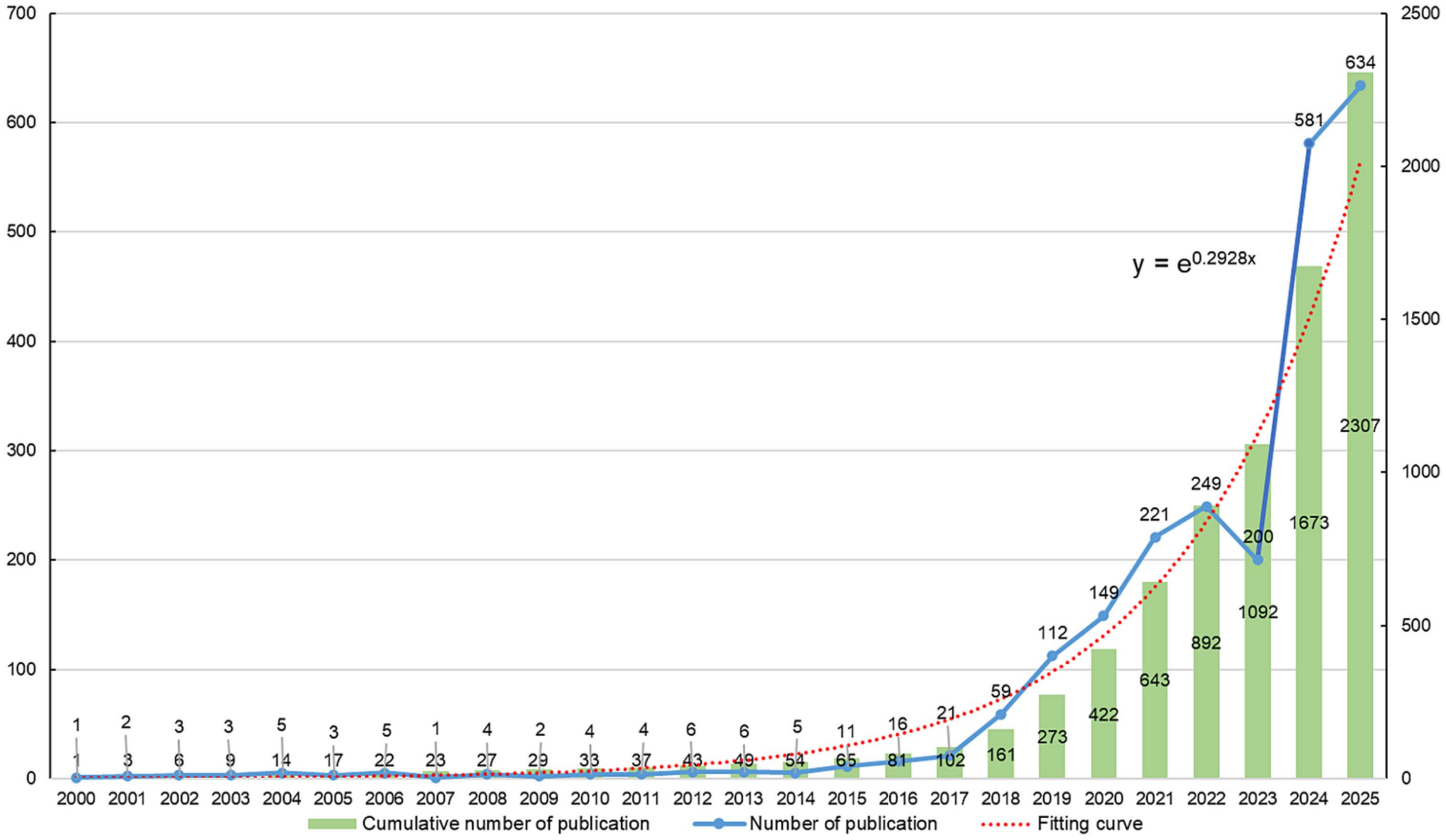
Annual publication output and cumulative growth of HFNO research (2000–2025). The figure shows annual publication output, cumulative number of publications, and the fitted exponential growth trend. HFNO research remained limited in the early years, then expanded rapidly after 2018, with a further marked increase after 2020.

The annual publication output and cumulative growth of HFNO research are shown in Figure 2. The field remained small during the early stage of development and expanded substantially in later years. Between 2000 and 2014, annual output remained low, ranging from 1 to 6 publications per year, with a cumulative total of 54 papers by the end of 2014. Output then increased gradually, reaching 11 publications in 2015, 16 in 2016, and 21 in 2017. After 2018, publication activity increased markedly. The annual number of publications rose from 59 in 2018 to 112 in 2019 and 149 in 2020, followed by 221 in 2021 and 249 in 2022. Although output declined slightly to 200 papers in 2023, it increased sharply to 581 in 2024 and 634 in 2025. Over the same period, the cumulative number of publications increased from 161 in 2018 to 2,307 by the end of 2025. These findings indicate an overall exponential growth pattern in HFNO research. The marked increase after 2020 coincided with the COVID-19 pandemic, during which HFNO received substantially greater clinical and research attention.

### 3.3 Journal landscape and core sources

**Figure 3:**
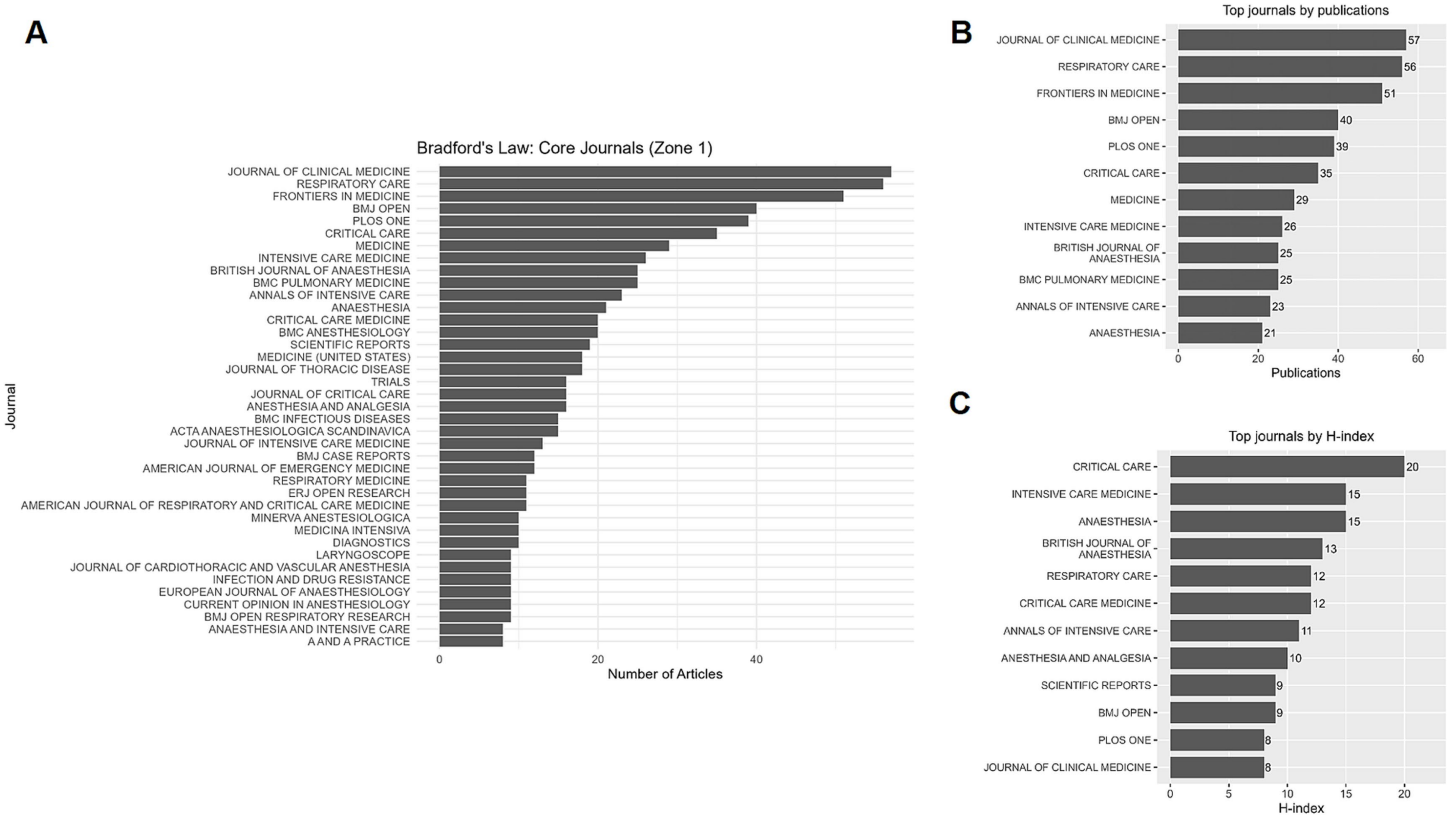
Journal landscape and core sources in HFNO research. **(A)** Core journals identified in Zone 1 according to Bradford’s law. **(B)** Top journals ranked by number of publications. **(C)** Top journals ranked by H-index within the retrieved dataset.

The journal distribution shown in Figure 3 indicates that HFNO research is concentrated in respiratory medicine, intensive care, and anesthesiology, with additional representation in general medical journals. According to Bradford’s law, a set of core journals was identified in Zone 1 (Figure 3A). In the output ranking (Figure 3B), Respiratory Care contributed the largest number of papers, followed by journals such as Journal of Clinical Medicine, Frontiers in Medicine, BMJ Open, and several critical care-oriented sources.

The citation-based ranking revealed a somewhat different pattern of influence. Highly cited HFNO studies were concentrated in leading critical care journals, and Figure 3C also presents journal H-index values calculated within the present dataset, allowing field-specific influence to be distinguished from journal-wide metrics. The top-ranked journals by this measure included Critical Care, Critical Care Medicine, and Anaesthesia. Overall, the journal analysis suggests two relatively consistent publication domains: respiratory support journals focused on clinical practice and critical care journals centered on trials, guidelines, and outcomes.

### 3.4 Global distribution and international collaboration patterns

**Figure 4:**
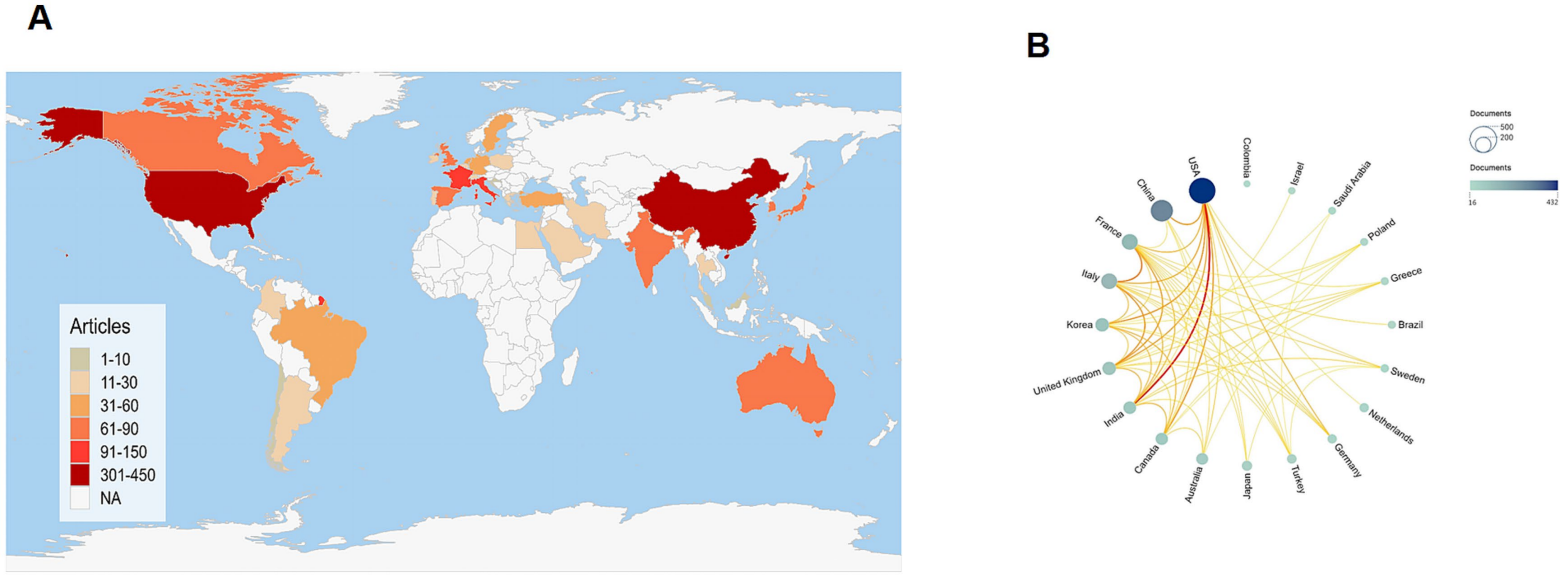
Global distribution and international collaboration of HFNO publications. **(A)** World map showing country-level publication output based on the merged dataset. **(B)** Country collaboration network generated by VOSviewer and refined for presentation; node size reflects publication volume, link thickness indicates collaboration strength, and colors represent clusters.

The geographic distribution of HFNO research (Figure 4A) demonstrates broad worldwide participation, although publication output was concentrated in a limited number of high-producing countries and regions. As shown in Table 1, the United States ranked first in publication output with 432 papers, followed by China with 363 papers. France and Italy formed the second tier, with 149 and 141 publications, respectively. Spain, Korea, the United Kingdom, India, Canada, and Australia also appeared among the top 10 contributors, indicating that HFNO research has been driven mainly by countries in North America, Europe, and the Asia-Pacific region.

**Table 1:** Top 10 countries by publication output in HFNO research.

Rank	Countries	Publications	Citations	Average citations per article
1	USA	432	5,321	12.30
2	China	363	2,801	7.70
3	France	149	2,554	17.10
4	Italy	141	3,093	21.90
5	Spain	86	1,094	12.70
6	Korea	82	500	6.10
7	United Kingdom	77	847	11.00
8	India	75	370	4.90
9	Canada	72	1,554	21.60
10	Australia	69	1,136	16.50

Citation indicators showed a pattern that was not entirely consistent with publication output. The United States ranked first in total citations [5,321], followed by Italy [3,093], China [2,801], and France [2,554]. In contrast, average citation impact was highest in Italy [21.9 citations per article], Canada [21.6], and France [17.1], whereas China and India showed lower average citation values despite substantial publication output. These findings suggest that research productivity and citation influence were related but not fully aligned across countries and regions.

The international collaboration network (Figure 4B) further indicates that the United States occupied the most central position in the global cooperation structure, with extensive links to multiple countries. China also formed a large and highly connected node, while France, Italy, the United Kingdom, Spain, and Canada were positioned within a closely linked collaboration cluster. Overall, the network suggests that HFNO research has developed through broad international cooperation but remains centered on a relatively small group of highly productive and well-connected countries. Taken together, these results show that the global HFNO literature is characterized by a combination of concentrated research output, uneven citation impact, and active cross-national collaboration.

### 3.5 Institutional productivity and collaboration network

**Figure 5:**
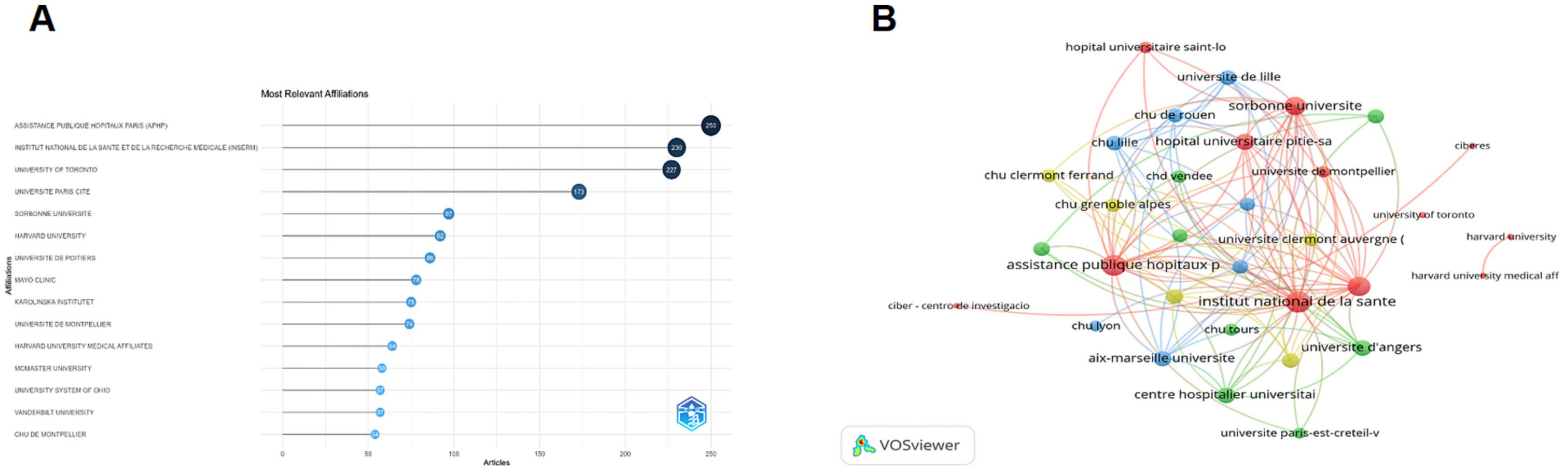
Institutional productivity and collaboration network in HFNO research. **(A)** Most relevant affiliations ranked by publication counts. **(B)** Institutional collaboration network generated by VOSviewer; node size reflects publication volume, link thickness indicates collaboration strength, and colors represent clusters.

The institutional distribution of HFNO research is presented in Figure 5. Assistance Publique Hôpitaux de Paris (APHP) ranked first with 250 publications, followed by the Institut National de la Santé et de la Recherche Médicale (INSERM) with 230 and the University of Toronto with 227. Université Paris Cité ranked fourth with 173 publications. Other productive institutions included Sorbonne Université [97], Harvard University [92], Université de Poitiers [86], Mayo Clinic [78], Karolinska Institutet [75], and Université de Montpellier [74].

The institutional collaboration network (Figure 5B) showed that several French institutions occupied central positions, particularly APHP, INSERM, Sorbonne Université, and Université de Montpellier. These institutions formed a dense collaboration cluster with affiliated hospitals and universities. By contrast, institutions such as the University of Toronto and Harvard University appeared less centrally positioned in the displayed network. Overall, HFNO research was concentrated in a relatively small number of highly productive academic and hospital-based institutions.

### 3.6 Author productivity, impact, and collaboration structure

**Figure 6:**
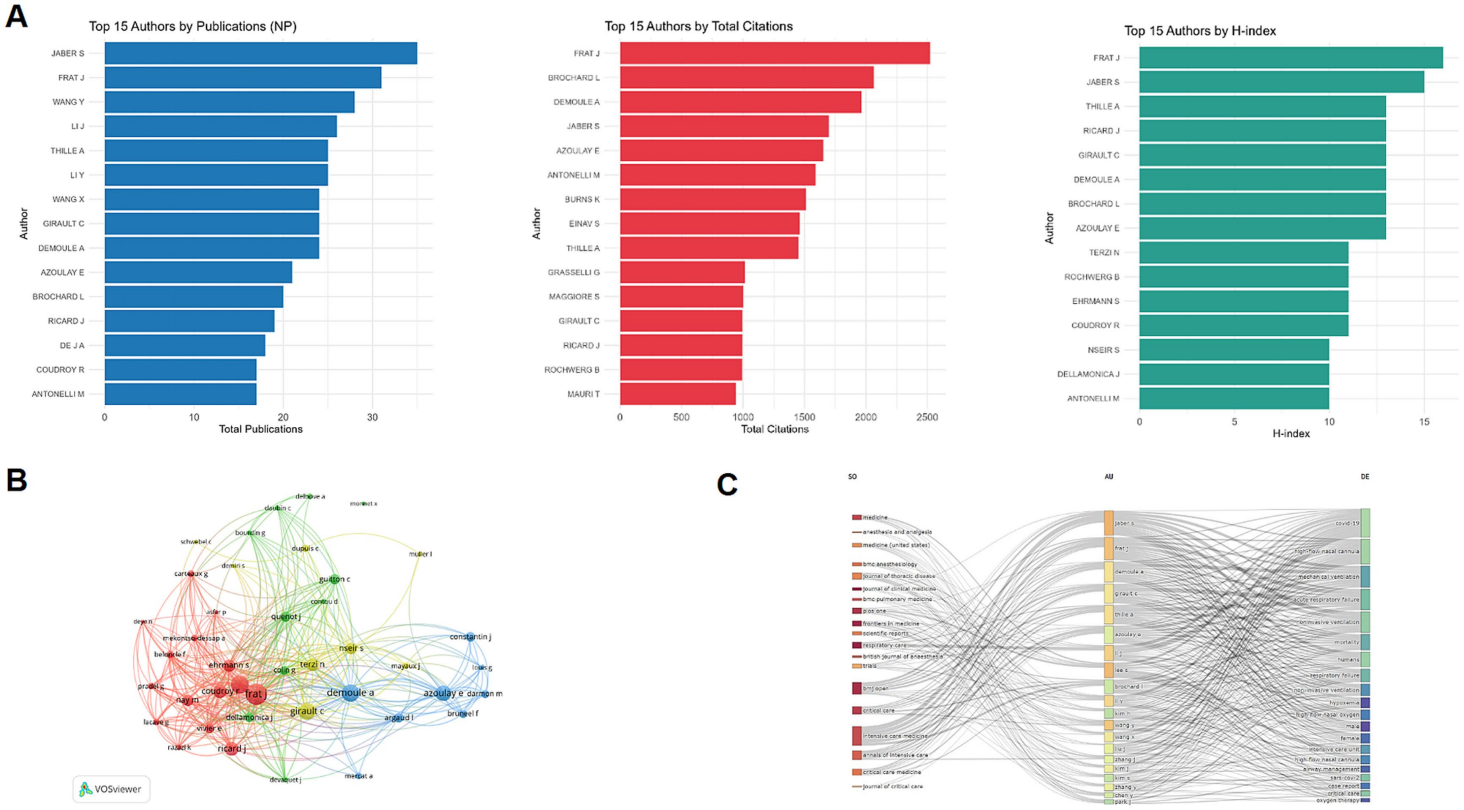
Author productivity, collaboration, and thematic linkages in HFNO research.**(A)** Top authors ranked by publications (NP), total citations (TC), and H-index (H). **(B)** Author collaboration network generated by VOSviewer; node size reflects publication volume and colors represent clusters. **(C)** Three-field plot linking sources (SO), authors (AU), and author keywords (DE); band width indicates the strength of association.

Author-level results are presented in Figure 6. In terms of publication output, Jaber S ranked first with 35 publications, followed by Frat J [31] and Wang Y [28]. By total citations, Frat J ranked first, followed by Brochard L and Demoule A. A similar pattern was observed for H-index, with Frat J ranking first and Jaber S second. These findings indicate that publication output and citation impact were related but not identical, although a small group of leading authors remained prominent across multiple indicators.

The co-authorship network (Figure 6B) revealed several closely connected collaboration clusters. One prominent group was centered on Frat J, with strong links to authors such as Ricard J and Coudroy R. Another major cluster was organized around Demoule A and Azoulay E. Taken together, the author network suggests that HFNO research has been shaped by several stable and well-connected collaborative teams.

The three-field plot linking sources, authors, and author keywords (Figure 6C) further illustrates the relationships among major publication outlets, leading contributors, and principal research topics. Several highly productive authors, including Jaber S, Frat J, Demoule A, Girault C, and Thille A, were closely associated with journals such as Critical Care, Intensive Care Medicine, Respiratory Care, and Journal of Clinical Medicine. On the keyword side, these authors were mainly linked to terms such as high-flow nasal cannula, acute respiratory failure, noninvasive ventilation, mechanical ventilation, and COVID-19. This pattern indicates that the core author group was closely connected to both intensive care-oriented clinical management and more recent pandemic-related research themes.

### 3.7 Co-citation structure, temporal evolution, and disciplinary citation paths

**Figure 7:**
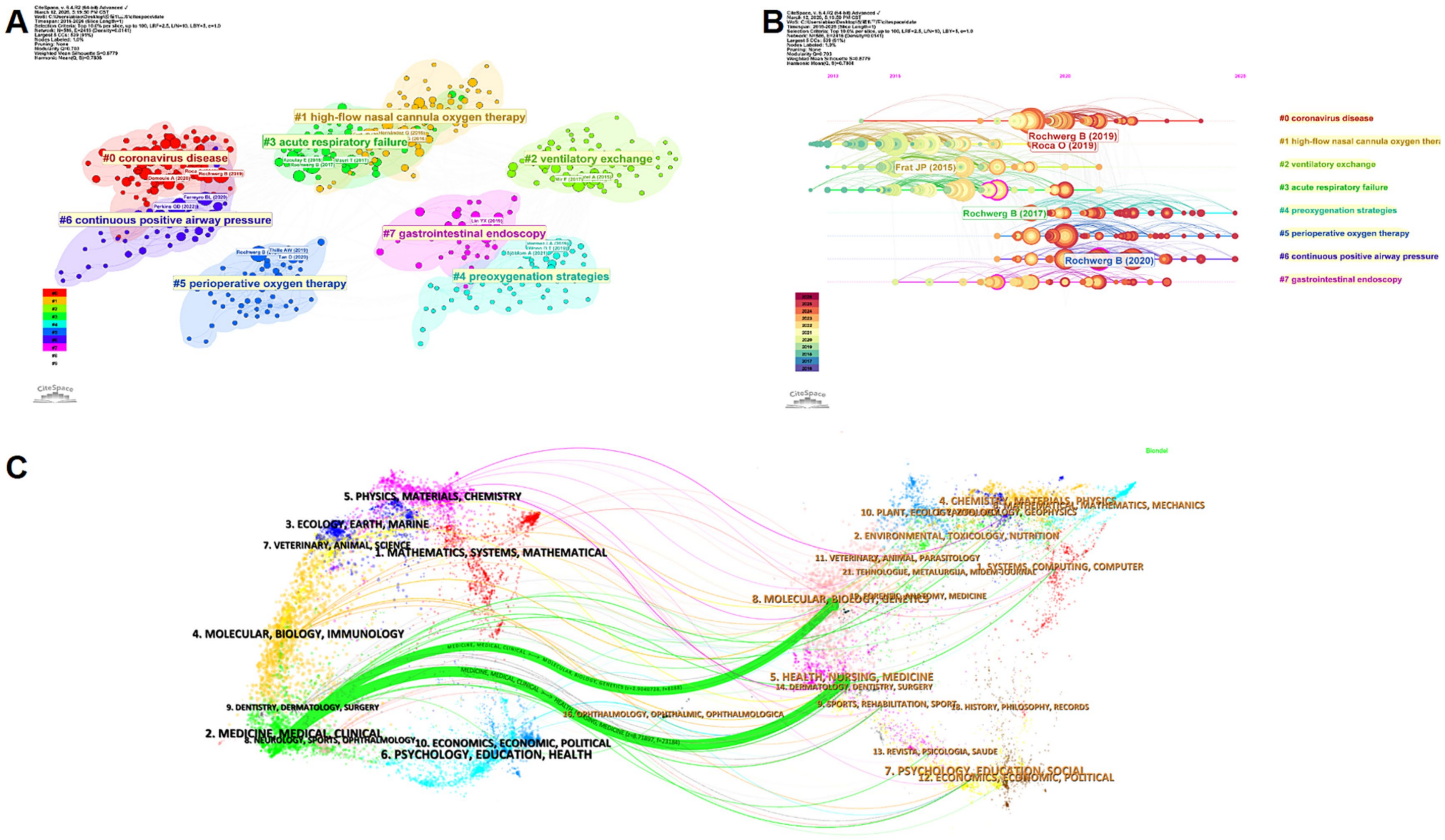
Co-citation structure, temporal evolution, and disciplinary citation paths in HFNO research. **(A)** Document co-citation network and major clusters generated by CiteSpace. **(B)** Timeline view of co-citation clusters showing their temporal evolution and key cited nodes. **(C)** Dual-map overlay of journals illustrating citation trajectories between citing and cited disciplines. CiteSpace-based analyses were restricted to the period 2015–2025. Together, these panels indicate that the intellectual base of HFNO research has shifted from earlier physiological and perioperative oxygenation topics toward acute respiratory failure, COVID-19-related care, and pathway-oriented clinical management.

The co-citation network and timeline view are shown in Figure 7. The co-citation network contained 586 nodes and 2,416 links (density = 0.0141), with a modularity Q value of 0.703 and a weighted mean silhouette value of 0.8779, indicating a well-structured and highly reliable clustering pattern. As shown in Figure 7A, the major co-citation clusters included #0 coronavirus disease, #1 high-flow nasal cannula oxygen therapy, #2 ventilatory exchange, #3 acute respiratory failure, #4 preoxygenation strategies, #5 perioperative oxygen therapy, #6 continuous positive airway pressure, and #7 gastrointestinal endoscopy. These clusters indicate that the intellectual structure of HFNO research spans critical care, perioperative airway management, and disease-specific clinical applications.

The timeline view (Figure 7B) further illustrates the temporal evolution of these co-citation clusters. Earlier influential references were concentrated in clusters related to ventilatory exchange, acute respiratory failure, and perioperative oxygen therapy, whereas later activity became more prominent in clusters such as coronavirus disease and high-flow nasal cannula oxygen therapy. Several highly influential references appeared as large and well-connected nodes across the timeline, including Frat JP [2015], Rochwerg B [2017, 2019, and 2020], and Roca O [2019]. This pattern indicates that a limited number of key clinical studies and evidence syntheses repeatedly shaped the development of the field. Overall, the timeline suggests a shift from earlier physiological and peri-intubation topics toward acute respiratory failure management and COVID-19-related clinical use.

The dual-map overlay (Figure 7C) shows the major citation paths of HFNO-related studies across disciplines. The citing journals were concentrated mainly in medicine, medical, and clinical fields, whereas the cited journals were distributed primarily in health, nursing, medicine, and molecular biology and genetics. The dominant citation paths indicate that HFNO research in clinical medicine has drawn heavily on both clinically oriented knowledge and broader biomedical foundations. This finding highlights the interdisciplinary character of the field and the close linkage between clinical application and underlying biological and medical research.

### 3.8 Keyword clusters, temporal evolution, and burst detection

**Figure 8:**
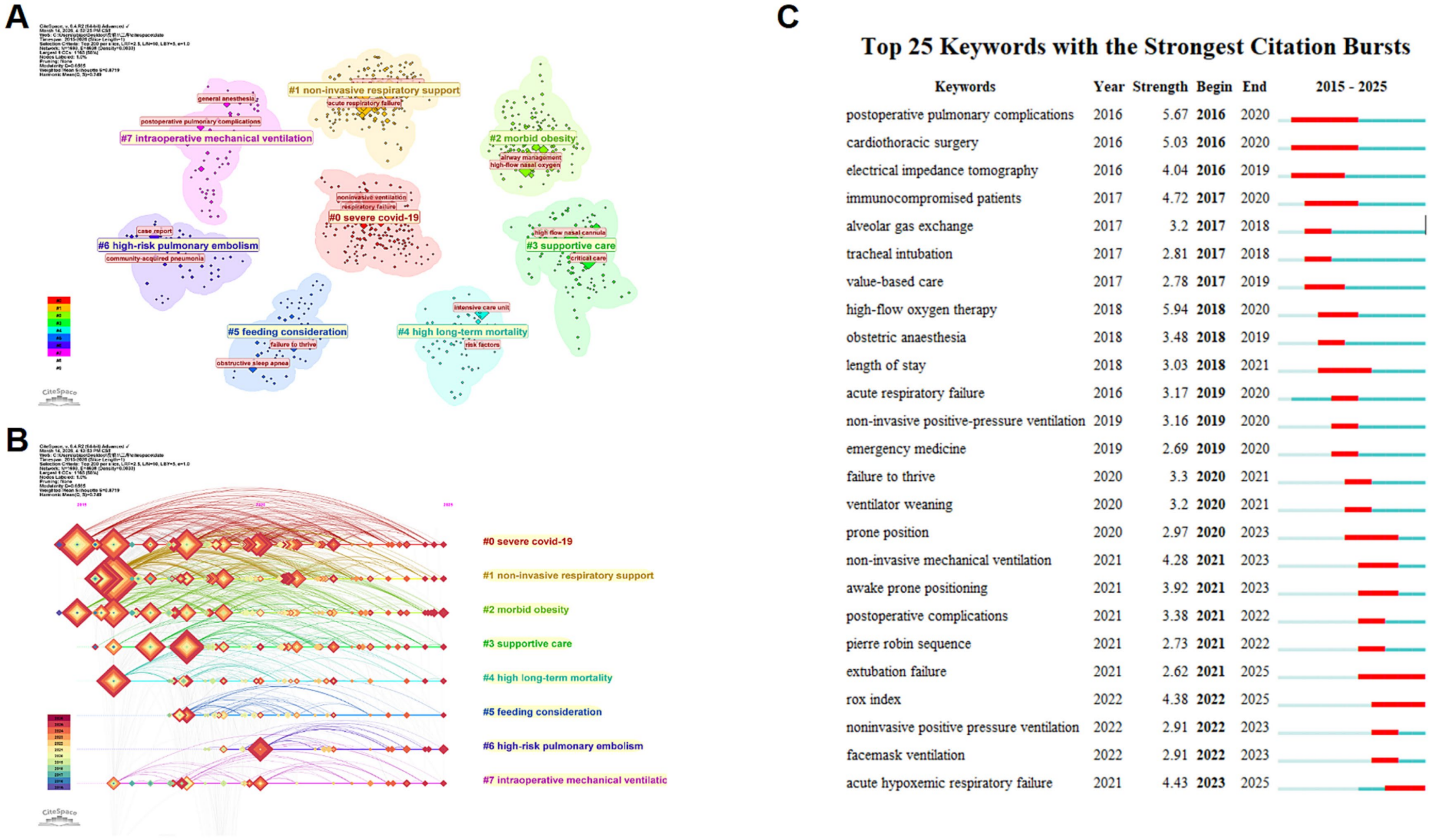
Keyword clusters, temporal evolution, and emerging fronts in HFNO research. **(A)** Keyword clustering map generated by CiteSpace. **(B)** Timeline view of keyword clusters showing the temporal shift of major research topics. **(C)** Top 25 keywords with the strongest bursts identified by CiteSpace. CiteSpace-based analyses were restricted to the period 2015–2025. The figure highlights the transition from perioperative oxygenation and procedure-related applications toward critical care use, failure prediction, awake prone positioning, and escalation-related decision-making.

Keyword analysis based on CiteSpace is shown in Figure 8. The clustering map (Figure 8A) identified several major thematic groups including #0 severe COVID-19 #1 non-invasive respiratory support #2 morbid obesity #3 supportive care #4 high long-term mortality and #7 intraoperative mechanical ventilation. These clusters indicate that HFNO research during the most active phase of the field was organized mainly around critical care respiratory support COVID-19-related management and perioperative applications

The timeline view (Figure 8B) showed a clear temporal shift in research focus. Earlier activity was more closely associated with perioperative and procedure-related topics, whereas later activity became increasingly concentrated in clusters related to severe COVID-19, non-invasive respiratory support, and supportive care. At the same time, later-stage keyword clusters also pointed to more specific clinical scenarios, including morbid obesity and intraoperative mechanical ventilation. This pattern suggests that HFNO research gradually expanded from general respiratory support to more targeted applications in defined patient groups and clinical settings.

Burst detection analysis (Figure 8C) provided further evidence of this transition. Early burst terms included oxygen therapy, cardiothoracic surgery, and postoperative pulmonary complications, reflecting an initial emphasis on perioperative respiratory support. More recent burst terms included awake prone positioning, high-flow nasal oxygenation, acute hypoxemic respiratory failure, and ROX index, indicating increasing attention to disease severity assessment, treatment escalation, and decision-making during HFNO therapy in acute care settings.

Overall, the keyword results indicate that HFNO research evolved from an early focus on perioperative techniques and postoperative complications toward broader use in critical care, followed more recently by targeted applications in specific clinical settings and increasing emphasis on escalation assessment and monitoring.

### 3.9 Thematic structure and thematic evolution

**Figure 9:**
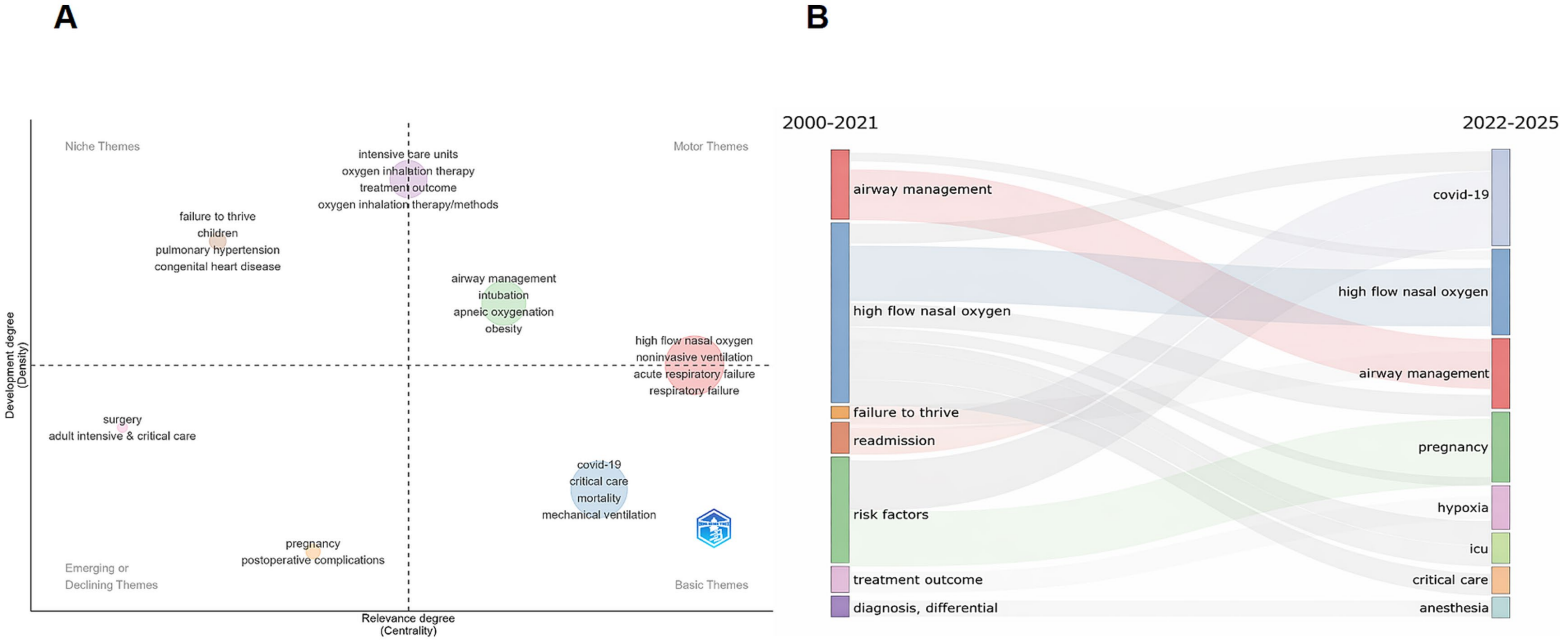
Thematic structure and thematic evolution of HFNO research. **(A)** Thematic map positioning topics by centrality and density, categorized into motor themes, basic themes, niche themes, and emerging or declining themes. **(B)** Thematic evolution across the defined time slices, showing continuity and transitions of major research themes over time.

The thematic map and thematic evolution analysis are shown in Figure 9. In the thematic map (Figure 9A), high flow nasal oxygen, noninvasive ventilation, and respiratory failure were located in the motor themes quadrant, indicating that these topics were both well developed and highly central to the field. Airway management, intubation, and obesity also appeared in the upper-right quadrant, suggesting that they represented important and relatively mature themes. By contrast, COVID-19, mortality, and intensive care unit were positioned in the basic themes quadrant, indicating high relevance to the field but comparatively lower internal development. Topics such as hypoxia, respiratory insufficiency, and intensive care units were located near the center of the map, reflecting a transitional position between centrality and density. In the niche themes quadrant, pneumothorax and septic shock appeared as more specialized topics, whereas anesthesia, pregnancy, and ICU were located in the emerging or declining themes quadrant.

The thematic evolution plot (Figure 9B) further illustrates how the thematic structure changed over time. From 2000–2021 to 2022–2025, high flow nasal oxygen remained a continuous core theme, and airway management also showed thematic continuity across the two periods. At the same time, the later period showed the emergence or increasing prominence of themes such as COVID-19, hypoxia, ICU, critical care, anesthesia, and pregnancy. Overall, these findings suggest that HFNO research has retained a stable core around oxygenation and airway management while progressively expanding into critical care, disease-specific applications, and selected perioperative or special-population settings.

## 4 Discussion

### 4.1 Principal findings and overall field profile

Taken together, the present findings suggest that HFNO research has moved from an early expansion phase to a more mature stage of development. Publication output remained low for many years, increased markedly after 2018, and accelerated further after 2020. This growth was accompanied by greater structural consolidation rather than by volume alone. A relatively stable group of respiratory, critical care, anesthesiology, and general clinical journals now anchors the literature. Influential output is concentrated in a limited number of countries, institutions, and collaborative teams, while the thematic core remains centered on high-flow nasal oxygen, noninvasive ventilation, respiratory failure, and airway management. Around this core, newer themes such as COVID-19, acute hypoxemic respiratory failure, supportive care, obesity, and perioperative applications have become increasingly visible. Overall, the field appears to be moving beyond broad technology adoption toward more specific questions of clinical positioning, including when HFNO is most useful, which patients are most suitable candidates, and when escalation should occur.

In this sense, the maturity of the HFNO field should be interpreted as clinical-pathway maturity rather than final evidentiary closure. The field has moved beyond the stage of asking whether high-flow oxygen can improve oxygenation or be tolerated by patients. It is now increasingly concerned with how HFNO should be positioned within a sequence of respiratory support decisions. This maturation has several dimensions. Methodologically, the literature has progressed from physiological observations and single-setting reports toward randomized trials, evidence syntheses, guideline-informed discussions, and comparative effectiveness studies. Clinically, the central question has shifted from whether HFNO works in general to which patients are most likely to benefit, how early response should be assessed, and when treatment failure should trigger escalation. Translationally, HFNO has entered routine bedside practice, but its use has not yet been stabilized into reproducible pathways across all settings.

This incomplete maturity is important. The remaining gaps are not simply gaps in publication volume, but gaps in decision quality. Patient selection remains inconsistent across perioperative, hypoxemic, post-extubation, COVID-19-related, and special-population settings. Outcome definitions also vary, with studies emphasizing intubation, mortality, oxygenation, comfort, ICU length of stay, or postoperative complications. Failure prediction remains unsettled: the ROX index has become a useful symbol of the field’s move toward dynamic monitoring, but its thresholds and timing are not universally transferable across diseases and care environments. The comparison between HFNO, CPAP, non-invasive ventilation, and invasive ventilation also remains context dependent. As a result, the main paradigm shift suggested by our findings is not simply from “less evidence” to “more evidence,” but from device adoption to decision architecture. Future HFNO research will need to define not only whether HFNO is effective, but how it should be embedded in a pathway that specifies patient selection, reassessment intervals, failure criteria, and escalation thresholds. Without this pathway perspective, HFNO may be continued too long in patients who are failing treatment, whereas overly early escalation may expose other patients to unnecessary invasive support.

### 4.2 COVID-19 accelerated expansion without redefining the core

The publication trajectory, keyword clusters, and thematic evolution all show a strong COVID-19 imprint, but they do not indicate that the pandemic replaced the pre-existing agenda of the field. Instead, COVID-19 appears to have accelerated ongoing development by increasing publication output and bringing topics such as severe COVID-19, supportive care, and ICU-related management to the foreground. At the same time, the thematic map continued to place high-flow nasal oxygen, noninvasive ventilation, and respiratory failure in the motor-theme quadrant, indicating that the conceptual center of the field remained relatively stable.

Before the pandemic, HFNO had already been studied in acute respiratory failure, peri-intubation oxygenation, and postoperative respiratory support. COVID-19 did not create these questions, but it sharply increased their clinical urgency. It also intensified attention to treatment monitoring, delayed intubation, awake prone positioning, and comparison with other non-invasive respiratory support strategies (23–26). COVID-era observational data further extended this discussion to real-world HFNO pathways and failure prediction (27).

The co-citation structure also suggests that the knowledge base of HFNO research has become broader and more layered. Earlier work was more closely linked to physiological support, ventilatory exchange, perioperative oxygen therapy, and preoxygenation. In the current maps, however, the major clusters extend into severe COVID-19, non-invasive respiratory support, supportive care, and several disease- or scenario-specific applications. This pattern suggests that the field has moved beyond proof-of-concept and beyond predominantly physiological explanation. Increasingly, the literature is concerned with how HFNO should be used across different treatment pathways, disease contexts, and clinical environments.

The timeline view supports this interpretation. A relatively small number of landmark studies remained highly influential across multiple clusters and periods, indicating that the field still relies on a durable evidence core. What has changed is the clinical context in which this evidence is applied. The same core studies are now interpreted within a broader landscape that includes acute respiratory failure, ICU workflows, COVID-era respiratory support, perioperative management, and selected procedure-related settings. The field therefore appears more differentiated than before, while still retaining a recognizable clinical center.

### 4.3 Perioperative and critical care integration remains a defining feature

One of the more notable findings of the present study is that perioperative HFNO remains structurally important rather than merely historical. In the thematic map, airway management, intubation, and obesity appear in the motor-theme region, indicating that these topics remain both central and relatively mature. Early burst terms such as cardiothoracic surgery, postoperative pulmonary complications, and apnoeic oxygenation also point to a strong perioperative and anesthesia-related foundation. In addition, both the co-citation structure and the keyword clusters retain clear procedure-related and intraoperative elements.

These findings suggest that perioperative HFNO should not be regarded simply as an earlier phase that has been displaced by ICU-focused research. Instead, perioperative and airway-management questions remain embedded within the active core of the field. Clinically, this is plausible because HFNO is particularly attractive in settings where oxygenation must be maintained while airway manipulation is minimized, spontaneous breathing is preserved, or peri-intubation safety is improved. The continued prominence of airway management and obesity further suggests that HFNO remains relevant in high-risk perioperative and peri-intubation populations, rather than only in generalized respiratory failure.

The thematic structure also points to a gradual shift toward more selective and context-specific use. Beyond the central themes of the field, the newer maps highlight topics such as obesity, pregnancy, critical care, ICU, and anesthesia. Even when some of these topics have not yet become fully developed motor themes, their appearance in the thematic evolution plot suggests that HFNO research is increasingly extending into more clearly defined patient groups and clinical contexts.

This trend may reflect maturation through specialization. In the early development of a field, research often asks whether a technique has broad value. At a later stage, the more clinically relevant question is where its value is greatest and where its limitations matter most. The present findings suggest that HFNO research is increasingly moving in this direction. Obese patients, airway-management settings, acute hypoxemic respiratory failure, ICU care, and selected perioperative or special-population scenarios appear to be emerging as key areas for refinement. Future progress may therefore depend less on indiscriminate expansion of use and more on defining appropriate clinical niches.

### 4.4 From device-centered comparison to pathway-centered management

Another clear signal from the present results is a shift away from device-centered comparison and toward pathway-centered management. Earlier burst terms largely reflected perioperative oxygenation techniques and postoperative respiratory support. More recent burst terms, including awake prone positioning, acute hypoxemic respiratory failure, high-flow nasal oxygenation, and ROX index, point to a different clinical problem: how HFNO should be monitored, when it should be continued, and when escalation should occur. In this sense, HFNO should not be viewed only as an oxygenation strategy, but as one component of a broader respiratory support pathway.

This shift is closely related to landmark clinical evidence and guideline-informed practice. The FLORALI trial helped establish HFNO as an important option in acute hypoxemic respiratory failure and stimulated broader interest in its role beyond simple oxygen delivery (3). Subsequent guidelines further placed HFNO within a wider noninvasive respiratory support framework, alongside conventional oxygen therapy, CPAP, non-invasive ventilation, and invasive mechanical ventilation (2). These developments help explain why recent HFNO research has increasingly focused on monitoring, treatment failure, and escalation decisions rather than on device feasibility alone.

The clinical meaning of this shift is that HFNO is no longer being evaluated as a stand-alone intervention in a uniform patient population. Its value depends strongly on context. In some settings, HFNO may be used as an initial strategy for *de novo* hypoxemic respiratory failure; in others, it may be applied after extubation, during peri-intubation management, in COVID-19-related respiratory failure, or in selected perioperative populations. This heterogeneity makes the comparison between HFNO and non-invasive ventilation difficult to reduce to a single conclusion. Trials and comparative studies, including recent work such as RENOVATE, have helped clarify parts of this question, but they also show that the relative value of HFNO varies according to patient phenotype, comparator, endpoint, and escalation strategy (23–25, 28). The more useful question is therefore not whether HFNO is globally superior or inferior to non-invasive ventilation, but where it fits best within a specific clinical pathway.

Failure prediction has become central to this pathway-based view. The ROX index is a clear example of how the field has moved from static device comparison toward dynamic reassessment during treatment (29, 30). Its prominence in the keyword and burst analyses is therefore clinically meaningful. However, ROX-based assessment is not a universal solution. Its performance may vary with the timing of measurement, disease severity, patient population, and local thresholds for intubation or non-invasive support. The emergence of multiple prediction tools and modified thresholds suggests that HFNO failure cannot be captured fully by a single score. Prediction tools are most useful when they are connected to a predefined action plan, rather than used as isolated numerical markers.

This issue is closely linked to the concern about delayed intubation. HFNO can improve comfort and short-term oxygenation, which partly explains its wide uptake, but these same features may also make treatment failure less obvious if reassessment is not structured. Continuing HFNO in a patient who is failing therapy may delay necessary intubation, whereas escalating too early may expose other patients to avoidable invasive support. The central challenge is therefore not simply whether HFNO should be used, but how clinicians should recognize response, define failure, and act on deterioration. Future studies should move toward pathway-based designs that specify patient selection, reassessment intervals, failure criteria, and escalation thresholds. Such designs would better reflect how HFNO is used at the bedside and would help move the field from bibliometric visibility toward clinically actionable decision-making.

### 4.5 Concentrated research structure, strengths, and limitations

The country, institution, and author analyses show a clear concentration of HFNO research, but the meaning of this pattern should be kept modest. In bibliometric terms, these maps mainly indicate where indexed publications, citations, and collaborations are most visible. They do not by themselves prove that particular countries, institutions, or author groups have greater clinical authority, nor do they show that their patterns of HFNO use are generalizable to all healthcare systems. The prominent positions of the United States, China, and several European research networks likely reflect a combination of publication volume, research capacity, English-language indexing, trial infrastructure, and database coverage. Their central positions should therefore be read as signals of publication capacity and collaboration density in the retrieved literature, rather than as a complete picture of global HFNO practice.

This concentration is still informative. HFNO is not only a device but also a bedside strategy that depends on monitoring resources, staffing, escalation culture, and access to intensive care. Large academic hospitals and well-connected research networks are therefore more likely to produce trials, guidelines, and evidence syntheses that shape the literature. At the same time, this means that highly visible evidence may be influenced by the conditions of relatively well-resourced systems. The next stage of HFNO research should therefore not only increase the number of publications, but also test HFNO-related pathways across a wider range of clinical environments.

This study has several strengths. By combining performance analysis, collaboration mapping, co-citation analysis, keyword evolution, and thematic mapping, it characterizes HFNO research from multiple complementary perspectives rather than from a single metric. These analytic layers point to a consistent overall pattern: HFNO research has developed a stable conceptual core around respiratory support and airway management, while its active questions are moving toward monitoring, failure prediction, escalation decisions, and selected patient groups.

Several limitations should also be acknowledged. The results depend on the quality and completeness of bibliographic metadata, database coverage, language restrictions, and the normalization of author, institution, country, and keyword fields. Although three databases were combined and duplicate records were removed, residual inconsistencies may remain. Citation-based indicators should also be interpreted cautiously because citations reflect visibility and time since publication, not necessarily methodological quality, clinical effect size, or patient benefit. Older trials, established authors, and long-standing institutions have more opportunity to accumulate citations. In addition, some CiteSpace analyses were restricted to 2015–2025 to improve interpretability during the most active phase of the field, which may underrepresent earlier signals. Thematic maps and keyword clusters should therefore be viewed as structured approximations of intellectual development, rather than direct measures of clinical validity.

Despite these limitations, the findings remain clinically informative when interpreted within these boundaries. HFNO research appears to have moved from broad adoption and oxygenation-focused application toward a more differentiated phase centered on patient selection, response monitoring, and escalation decisions. This evolution, rather than the dominance of any specific country or institution, is likely to define the next stage of HFNO research.

## Data Availability

The raw data supporting the conclusions of this article will be made available by the authors, without undue reservation.
