# Supplementary material for: High-flow nasal oxygen in perioperative and critical care: a bibliometric analysis
Source: Front Med (Lausanne). 2026 Jul 15;13:1856380. doi: 10.3389/fmed.2026.1856380 (PMC13415779; doi:10.3389/fmed.2026.1856380)
Supplement: Supplementary file 1 [file Table_1.docx]

WoSCC：TS=("high flow nasal oxygen" OR "high-flow nasal oxygen" OR HFNO OR "high flow nasal cannula*" OR "high-flow nasal cannula*" OR HFNC OR THRIVE OR "transnasal humidified rapid-insufflation ventilatory exchange" OR "humidified high flow" OR "heated humidified high flow") AND TS=(perioperat* OR anesthe* OR anaesthe* OR surgery OR surgical OR "airway management" OR apneic OR apnea OR "apnoeic" OR ICU OR "intensive care" OR "critical care") NOT TS=(neonat* OR pediatric* OR paediatr*)

Scopus：TITLE-ABS-KEY("high flow nasal oxygen" OR "high-flow nasal oxygen" OR HFNO OR "high flow nasal cannula*" OR "high-flow nasal cannula*" OR HFNC OR THRIVE OR "transnasal humidified rapid-insufflation ventilatory exchange" OR "humidified high flow" OR "heated humidified high flow") AND TITLE-ABS-KEY(perioperat* OR anesthe* OR anaesthe* OR surgery OR surgical OR "airway management" OR apneic OR apnea OR "apnoeic" OR ICU OR "intensive care" OR "critical care") NOT TITLE-ABS-KEY(neonat* OR pediatric* OR paediatr*) AND PUBYEAR > 1999 AND PUBYEAR < 2026

PubMed：("high flow nasal oxygen"[Title/Abstract] OR "high-flow nasal oxygen"[Title/Abstract] OR HFNO[Title/Abstract] OR "high flow nasal cannula*"[Title/Abstract] OR "high-flow nasal cannula*"[Title/Abstract] OR HFNC[Title/Abstract] OR THRIVE[Title/Abstract] OR "transnasal humidified rapid-insufflation ventilatory exchange"[Title/Abstract] OR "humidified high flow"[Title/Abstract] OR "heated humidified high flow"[Title/Abstract]) AND ("perioperat*"[Title/Abstract] OR anesthe*[Title/Abstract] OR anaesthe*[Title/Abstract] OR surgery[Title/Abstract] OR surgical[Title/Abstract] OR "airway management"[Title/Abstract] OR apneic[Title/Abstract] OR apnea[Title/Abstract] OR "apnoeic"[Title/Abstract] OR ICU[Title/Abstract] OR "intensive care"[Title/Abstract] OR "critical care"[Title/Abstract]) NOT ("neonat*"[Title/Abstract] OR pediatric*[Title/Abstract] OR paediatr*[Title/Abstract]) AND ("2000/01/01"[Date - Publication] : "2025/12/31"[Date - Publication])
